# Clinical and microbiological characteristics of cystic fibrosis adults never colonized by *Pseudomonas aeruginosa*: Analysis of the French CF registry

**DOI:** 10.1371/journal.pone.0210201

**Published:** 2019-01-08

**Authors:** Réchana Vongthilath, Bénédicte Richaud Thiriez, Clémence Dehillotte, Lydie Lemonnier, Alicia Guillien, Bruno Degano, Marie-Laure Dalphin, Jean-Charles Dalphin, Patrick Plésiat

**Affiliations:** 1 Department of Respiratory Medicine, University Hospital Jean Minjoz, Besançon, France; 2 Medical Department of Vaincre La Mucoviscidose, Paris, France; 3 Department of Physiology, University Hospital Jean Minjoz, Besançon, France; 4 EA3920, University of Franche-Comté, Besançon, France; 5 Department of Pediatric Medicine, University Hospital Jean Minjoz, Besançon, France; 6 UMR/CNRS 6249 Chrono-Environnement, University of Franche-Comté, Besançon, France; 7 Department of Bacteriology, University Hospital Jean Minjoz, Besançon, France; Ohio State University, UNITED STATES

## Abstract

*Pseudomonas aeruginosa* is the main cause of chronic airway infection in cystic fibrosis (CF). However, for unclear reasons some patients are never colonized by *P*. *aeruginosa*. The objectives of this study were to better define the clinical, genetic, and microbiological characteristics of such a subpopulation and to identify predictive factors of non-colonization with *P*. *aeruginosa*. The French CF patient registry 2013–2014 was used to identify CF patients aged ≥ 20 years. The clinical outcomes, CF Transmembrane conductance Regulator (CFTR) genotypes, and microbiological data of patients reported positive at least once for *P*. *aeruginosa* (“Pyo” group, *n* = 1,827) were compared to those of patients with no history of *P*. *aeruginosa* isolation (“Never” group, *n* = 303). Predictive factors of non-colonization by *P*. *aeruginosa* were identified by multivariate logistic regression model with backward selection. Absence of aspergillosis (odds ratio (OR) [95% CI] = 1.64 [1.01–2.66]), absence of diabetes (2.25 [1.21–4.18]), pancreatic sufficiency (1.81 [1.30–2.52]), forced expiratory volume 1 (FEV1) ≥ 80% (3.03 [2.28–4.03]), older age at CF diagnosis (1.03 [1.02–1.04]), and absence of F508del/F508del genotype (2.17 [1.48–3.19]) were predictive clinical factors associated with absence of infection (“Never” group). Microbiologically, this same group was associated with more frequent detection of *Haemophilus influenzae* and lower rates of *Stenotrophomonas maltophilia*, *Achromobacter xylosoxidans* and *Aspergillus spp*. (all *p*<0.01) in sputum. This study strongly suggests that the absence of pulmonary colonization by *P*. *aeruginosa* in a minority of CF adults (14.2%) is associated with a milder form of the disease. Recent progress in the development of drugs to correct CFTR deficiency thus may be decisive in the control of *P*. *aeruginosa* lung infection.

## Introduction

Cystic fibrosis (CF), the most common inherited disease in Caucasian populations is due to alteration of the *CFTR* gene [1]. Among the many mutations reported to date known to impact the activity of the encoded chloride channel CFTR (CF Transmembrane conductance Regulator), deletion of the phenylalanine residue at position 508 (F508del) is of major clinical importance. This mutation is indeed associated with about 70 percent of defective *CFTR* alleles [2]. The impaired mucociliary clearance that results from CFTR dysfunction predisposes patients’ lungs to colonization by a variety of opportunistic pathogens including *Pseudomonas aeruginosa*. This Gram-negative environmental bacterium is a well-known cause of morbidity and mortality in CF patients [3–5]. Its occurrence in the airways elicits a chronic inflammation and recurrent pulmonary exacerbations that contribute to a progressive decline in respiratory function over time [6]. According to the European CF Society patient registry, chronic infection by *P*. *aeruginosa* increases the odds ratio of severe lung disease by 2.4 (95% CI 2.0–2.7) compared to non-colonized patients, after adjustment for age and other potential confounding factors [7].

Annual reports of national CF registries from Canada (https://www.cysticfibrosis.ca/), France (http://www.vaincrelamuco.org), Germany (http://www.cysticfibrosisdata.org/), Ireland (https://www.cfri.ie/index.php), United Kingdom (https://www.cysticfibrosis.org.uk/), and the USA (https://www.cff.org/) all indicate that the prevalence of *P*. *aeruginosa* in respiratory secretions gradually increases with patients’ age up to 20–25 years, and then tends to stabilize afterwards at around 50–70%. As such, these data seem to indicate that 30–50% of adult CF patients are free of *P*. *aeruginosa* colonization during the year the survey is made. While it is evident that some of these patients have been cleared from their infection by antibiotic treatments prior to the survey or are intermittently colonized by *P*. *aeruginosa* (with no positive sample recorded during the survey), some others may well have never been infected by *P*. *aeruginosa* during their lifetime. The present study was thus designed to better characterize this latter subpopulation of patients, by statistically exploiting the demographic, clinical and microbiological data of the French CF registry.

## Materials and methods

### Ethics statement

Statistical analyses on the French CF registry were performed on a fully anonymized database. No informed content of patients was required. Data cannot be shared publicy because they contain confidential information which is protected by patient privacy legislation. Researchers who meet the criteria for access to confidential data should contact the medical department of the French cystic fibrosis association VLM (Anne Farge MD, afarge@vaincrelamuco.org).

### Patient population

Data from the French CF registry were retrospectively analyzed. At the time of the study, a total of 6,412 patients were medically followed by 45 CF care centers (Centres de Ressources et de Compétences de la Mucoviscidose, CRCM) in France. Once a year, these centers report predefined demographic, diagnostic and therapeutic data to the national registry for statistical analyses. All patients aged ≥ 20 years, who were recorded in the database between January 1^st^ 2013 and December 31^st^ 2014, were considered for further investigations. As mentioned above, the cut-off age of 20 years was chosen because the prevalence of *P*. *aeruginosa* in sputum samples tends to stabilize in CF from this age onwards. Lung transplant patients and those with no microbiological follow-up during the study period were excluded.

### Target population analysis

The patients were assigned to two different groups according to their bronchopulmonary colonization status. A patient was classed in the “Pyo” group if she/he had been reported positive for *P*. *aeruginosa* at least once prior to the study period. Conversely, patients with no known history of *P*. *aeruginosa* since their inclusion in the database were classed in the “Never” group. All analyses compared these two groups. It should be mentioned here that CF patients are included in the CF registry at the time of their initial diagnosis.

### Clinical and microbiological data

The following variables were included in the descriptive analysis: age at the start of the study period, age at CF diagnosis, gender, body mass index (BMI), presence of F508del/F508del genotype, pancreatic insufficiency (yes or no) based on enzyme usage, diabetes (yes or no) based on the use of antidiabetic drugs, aspergillosis (yes or no) if treated by antifungals, respiratory function (forced expiratory volume in the first second (FEV1), forced vital capacity (FVC), and pulmonary complications such as pneumothorax or hemoptysis.

Microbiological methods for the detection and quantification of *P*. *aeruginosa*, *Haemophilus influenzae*, *Streptococcus pneumoniae*, *Staphylococcus aureus*, *Stenotrophomonas maltophilia*, *Burkholderia cepacia*, *Aspergillus spp* and *Achromobacter xylososidans* in respiratory samples were performed by the laboratories affiliated to CF care centers, according to French laboratory guidelines [8]. Analysis data are transmitted to the French registry annually.

### Statistical methods

Qualitative variables are presented as numbers and percentages, and quantitative variables as means ± standard deviations (SD). For quantitative values, the mean ± SD of the two years of the study period is presented. Missing data were not replaced. Comparisons between the “Pyo” and “Never” groups were performed and odds ratios (OR) were computed using logistic regression, with the SAS v9.4 software package. Factors with a *p*-value less than 0.05 in bivariate analysis were included in the multivariate logistic regression with a backward selection, and corresponding odds ratios were calculated with their 95% confidence intervals (95% CI). Identification of clinical and microbiological data were performed using two separate multivariate analyses.

## Results

**Fig 1:**
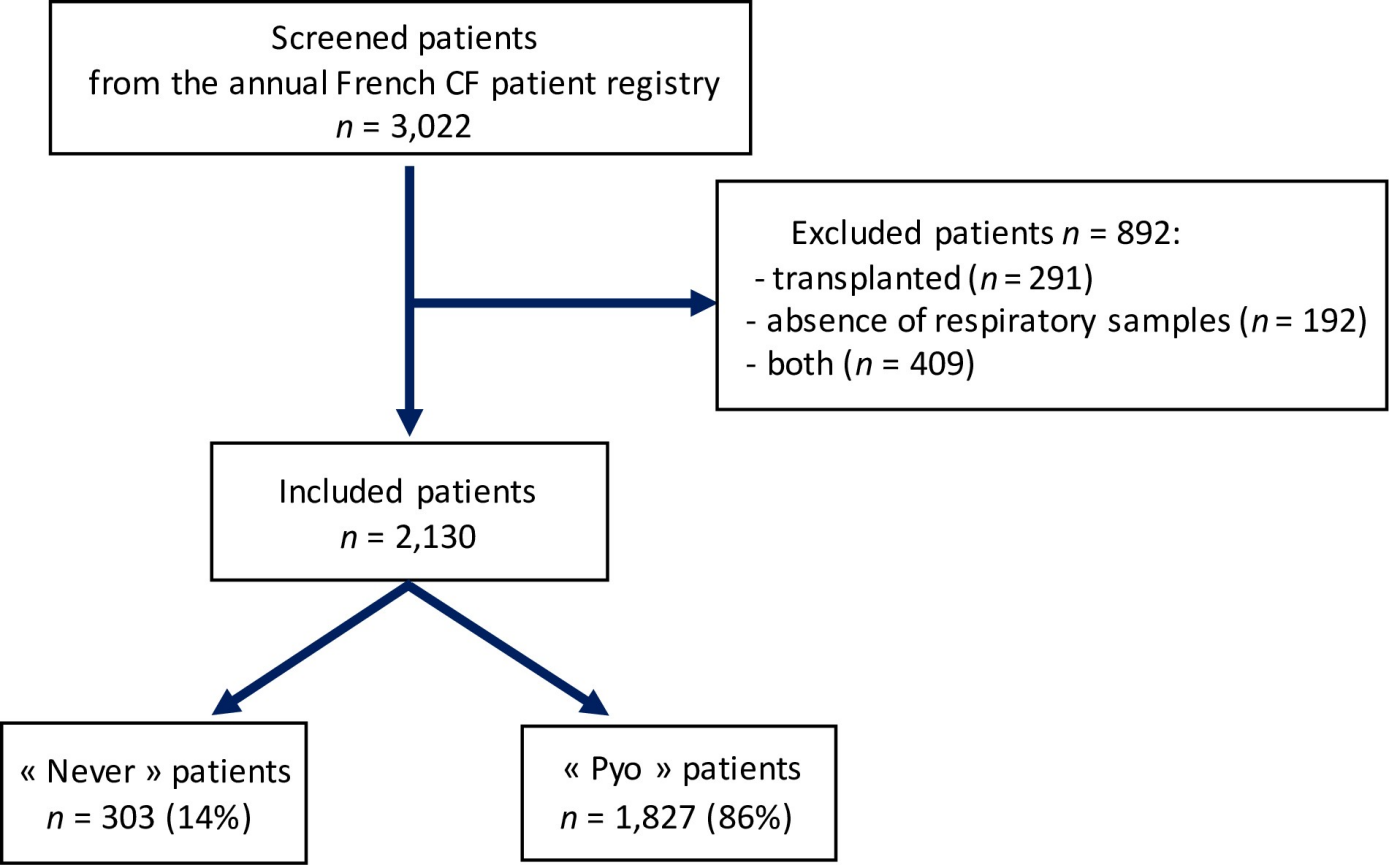
Flow chart of the population.

Among the 3,022 CF patients aged ≥ 20 years, 2,130 were included in the study. A total of 291 patients were excluded because of organ transplant, missing microbiological data (*n* = 192), or both (*n* = 409) (Fig 1). The excluded patients did not differ significantly from the included subjects in terms of age at CF diagnosis, gender, genotype, status of pancreatic function, and pneumothorax. On the other hand, excluded patients were older (34 years *vs* 32, *p* < 0.001), more often had diabetes (47% *vs* 17%, *p* < 0.001) and FEV1 ≥ 80% (49% *vs* 31%, *p* < 0.001). They also had fewer pulmonary complications such as hemoptysis and aspergillosis (3% *vs* 10%, *p* < 0.001 and 7% *vs* 15%, *p* < 0.001, respectively) than the included population.

The patients included were divided into two groups, namely the “Never” (*n* = 303; 14.2%) and “Pyo” groups (*n* = 1827; 85.8%). Table 1 shows the main demographic and clinical characteristics of both groups, which were not significantly different with respect to pulmonary complications (pneumothorax and hemoptysis) and gender. Conversely, bivariate analysis of clinical data indicated that “Never” patients had a less severe phenotype.

**Table 1:** Demographic and clinical characteristics of the study population.

	"Never"	"Pyo"	OR [95% CI]	*p*-value *
	*n* = 303	*n* = 1,827		
**Age in 2013** (years)	35 ± 13	31 ± 10	1.03 [1.02–1.04]	**< 0.001**
**Age at CF diagnosis** (years) (MD = 25–121)	20 ± 19	7 ± 12	1.05 [1.04–1.06]	**< 0.001**
**Male**, *n* (%)	167 (55)	962 (53)	1.10 [0.86–1.41]	0.43
**BMI** (kg/m^2^) (MD = 1–11)	23 ± 4	21 ± 3	1.12 [1.09–1.16]	**< 0.001**
**F508del/F508del genotype**, *n* (%)	50 (17)	812 (44)	0.25 [0.18–0.34]	**< 0.001**
**Pancreatic insufficiency**, *n* (%)	165 (55)	1552 (85)	0.21 [0.16–0.27]	**< 0.001**
**Diabetes**, *n* (%)	14 (5)	348 (19)	0.21 [0.12–0.36]	**< 0.001**
**Aspergillosis**, *n* (%)	23 (8)	288 (16)	0.44 [0.28–0.68]	**< 0.001**
**FVC ≥ 80% predicted** (MD = 7–17)	222 (75)	993 (55)	2.47 [1.87–3.26]	**< 0.001**
**FEV1 ≥ 80% predicted** (MD = 5–16)	165 (55)	480 (26)	3.44 [2.68–4.42]	**< 0.001**
**Pneumothorax**, *n* (%)	3 (1)	22 (1)	0.82 [0.24–2.76]	0.75
**Hemoptysis**, *n* (%)	23 (8)	183 (10)	0.74 [0.47–1.16]	0.19

Values are expressed as *n*, *n* (%) or mean ± standard deviation

* *p*-values correspond to logistic regressions’*p*-values

MD = x-y means there was x missing data in “Never” and y in “Pyo”

The microbiological features of the two groups are shown in Table 2. In contrast to *H*. *influenzae*, the environmental species *S*. *maltophilia*, *A*. *xylosoxidans*, and *Aspergillus spp* were less frequently isolated in the “Never” group than in the “Pyo” group (all *p* < 0.001), whereas *S*. *pneumoniae* and *S*. *aureus* tended to be over-represented in the “Never” group compared to the “Pyo” group (*p* < 0.20).

**Table 2:** Microbiological characteristics of the study population.

Microorganism		"Never" (*n* = 303)	"Pyo" (*n* = 1,827)	OR [95% CI]	*p*-value
***H*. *influenzae***					
	No	205 (67.7%)	1467 (80.3%)	1	-
	Yes	98 (32.3%)	360 (19.7%)	1.95 [1.49–2.54]	**< 0.001**
***S*. *pneumoniae***					
	No	294 (97%)	1796 (98.3%)	1	-
	Yes	9 (3%)	31 (1.7%)	1.77 [0.84–3.76]	0.14
***S*. *aureus***					
	No	74 (24.4%)	514 (28.1%)	1	-
	Yes	229 (75.6%)	1313 (71.9%)	1.21 [0.91–1.6]	0.18
***S*. *maltophilia***					
	No	279 (92.1%)	1522 (83.3%)	2.33 [1.51–3.60]	**< 0.001**
	Yes	24 (7.9%)	305 (16.7%)	1	-
***B*. *cepacia***					
	No	292 (96.4%)	1746 (95.6%)	1.23 [0.65–2.34]	0.53
	Yes	11 (3.6%)	81 (4.4%)	1	-
***Aspergillus spp***					
	No	198 (65.3%)	843 (46.1%)	2.20 [1.71–2.84]	**< 0.001**
	Yes	105 (34.7%)	984 (53.5%)	1	-
***A*. *xylosoxidans***					
	No	290 (95.7%)	1636 (89.5%)	2.60 [1.46–4.63]	**0.001**
	Yes	13 (4.3%)	191 (10.5%)	1	-

Values are expressed as *n* (%).

Logistic regression with backward selection indicated that the clinical variables predictive of non-colonization by *P*. *aeruginosa* in adult CF patients were: (i) absence of aspergillosis, (ii) older age at CF diagnosis, (iii) absence of diabetes, (iv) absence of pancreatic insufficiency, (v) absence of F508del homozygosity, and (vi) preserved FEV1 (Table 3). The presence of *H*. *influenza*e, the absence of *S*. *maltophilia*, *A*. *xylosoxidans*, and *Aspergillus spp*. were found to be associated with the absence of *P*. *aeruginosa* in group “Never” (Table 4).

**Table 3:** Multivariate analysis of clinical data associated with non-colonization by *P*. *aeruginosa*.

Clinical parameters	OR [95% CI]	*p*-value
Absence of aspergillosis	1.64 [1.01–2.66]	**0.0476**
Absence of diabetes	2.25 [1.21–4.18]	**0.01**
Absence of pancreatic insufficiency	1.81 [1.3–2.52]	**< 0.001**
FEV1 (%) ≥ 80% predicted	3.03 [2.28–4.03]	**< 0.001**
Absence of F508del homozygosity	2.17 [1.48–3.19]	**< 0.001**
Age at CF diagnosis	1.03 [1.02–1.04]	**< 0.001**

**Table 4:** Multivariate analysis of microbiological data associated with non-colonization by *P*. *aeruginosa*.

Microorganism		OR [95% CI]	*p*-value
*A*. *xylosoxidans* (absence)		0.39 [0.22–0.69]	**0.001**
*Aspergillus spp* (absence)		0.47 [0.36–0.6]	**< 0.001**
*H*. *influenzae* (presence)		2.18 [1.66–2.87]	**< 0.001**
*S*. *maltophilia* (absence)		0.44 [0.28–0.69]	**< 0.001**

## Discussion

To the best our knowledge, this is the first study to focus on the characteristics of CF adults with no known history of infection or colonization by *P*. *aeruginosa*. The prevalence of “Never” patients among the CF adult population was 14%, while national CF registries report up to 50% of patients negative for *P*. *aeruginosa*. Actually, these high rates include patients with intermittent colonization (*i*.*e*, found to be negative during the survey period) and those cleared from their infection by chemotherapy, in addition to patients who have “never” been infected (*i*.*e*., no respiratory sample with *P*. *aeruginosa* over the detection limit of 10^2^ CFU/mL).

Six clinical parameters were found to be independently associated with “Never” patients, including four comorbidities that can potentially be treated or prevented (aspergillosis, diabetes, pancreatic insufficiency and respiratory function impairment). A fifth factor related to the severity of CFTR dysfunction (FEV1) may partially be improved by novel targeted therapies [9].

Aspergillosis has previously been reported as a risk factor for lung colonization by *P*. *aeruginosa*. According to a retrospective case-control study, a longer duration of *P*. *aeruginosa* colonization was found to be independently associated with *A*. *fumigatus* sensitization (OR per year 1.50; 95% CI 1.12- infinity) [10]. A more recent work assessed mutual microbial interactions and analyzed the effects of bronchial colonization by a given microbial species on the risk of colonization by other microorganisms. The authors demonstrated that *A*. *fumigatus* was associated with an increased risk of *P*. *aeruginosa* acquisition [11]. A plausible explanation is the treatment of aspergillosis by glucocorticoids, which could impair internalization and phagocytosis of *P*. *aeruginosa* by CF respiratory epithelial cells [12].

CF-related diabetes and pancreatic insufficiency were associated with *P*. *aeruginosa* lung colonization likely because of their negative effects on the patient's condition, but also because they reflect disease severity. Indeed, the progression of FEV1 over time was found to be negatively impacted by diabetes or the pancreatic status of CF patients [7, 13, 14]. By temporarily improving pulmonary function [15], treatments with insulin or pancreatic extracts might delay *P*. *aeruginosa* acquisition in the airways.

Our study shows that the absence of F508del homozygosity is associated with a lower risk of colonization by *P*. *aeruginosa*. By extrapolation, and in agreement with Kubesh *et al*. [16], patients with a F508del/F508del genotype (almost 50% of the CF population in France; http://www.vaincrelamuco.org) thus appear to be more vulnerable than others to *P*. *aeruginosa*. This microorganism has been reported to adhere more strongly to respiratory epithelial cells from homozygous patients than to those from other genetic backgrounds [17]. Finally, some data support the hypothesis that the CFTR protein itself might serve as a cellular receptor for binding, endocytosis, and then clearance of *P*. *aeruginosa* [18, 19]. If this assumption is correct, any strong defect in CFTR, such as the one resulting from the F508del/F508del background, would impact this defense mechanism.

Older age at CF diagnosis, the last clinical factor associated with non-colonization by *P*. *aeruginosa*, could reflect a milder, pauci-symptomatic form of the disease, and better immune defenses to resist pulmonary invasion by the pathogen. Alternatively, the presence of *P*. *aeruginosa* could be a better alert for diagnosis of CF than CF symptoms and comorbidities. Since the systematic screening of newborns for CF was implemented in 2002, none of the adult patients of the study benefited from this test. Thus, it can be anticipated that the age factor will probably be less pertinent in the future for screened populations. The gender distribution was similar between the “Never” and “Pyo” groups despite a disadvantage for female patients, known to experience faster decline in FEV1 than male patients [20].

Backward logistic regression provided evidence that environmental microorganisms such as *S*. *maltophilia*, *A*. *xylosoxidans* and *Aspergillus spp* are less prevalent in the “Never” group than in the “Pyo” group. Their deleterious effects on pulmonary function in CF have been established [21–25]. However, the fact that these organisms, just like *P*. *aeruginosa*, are essentially responsible for opportunistic infections reinforce the idea that patients in the “Never” group have a better health status than those of the “Pyo” group. As suggested by Hector *et al*., the higher rates of *H*. *influenzae* colonization in the “Never” group could also reflect better lung function [21–25]. Although not statistically significant, the absence of *P*. *aeruginosa* in “Never” patients was associated with somewhat more frequent lung colonization by *S*. *aureus*, as noted previously [11].

CF registries are a valuable resource for research studies but have their own limitations. Indeed, a causal relationship between the factors identified and *P*. *aeruginosa* negative patients could not be established. A prospective study would be necessary to assess these factors, especially in the pediatric population, as the primary colonization with *P*. *aeruginosa* occurs at a mean age of 8 years in our country (http://www.vaincrelamuco.org).

Some variables were unavailable at the time of the study and will be soon captured in the French CF registry, such as the frequency of visits and the total number of sputum samples per year. Since the “Pyo” group referred to CF patients with one or more positive sputum cultures for *P*. *aeruginosa*, it consisted of patients with either intermittent colonization or chronic colonization. The colonization status (intermittent *versus* chronic) of “Pyo” patients according to Leeds criteria [26] could not be established because the French CF registry did not contain the appropriate microbiological data to enable such classification. Furthermore, the total number of sputum samples per year was not available. The features of adult patients previously colonized by *P*. *aeruginosa* but free of *P*. *aeruginosa* at the moment of the study would certainly be interesting to determine, as this population may be an intermediate stage between the “Never” and “Pyo” groups. Standardization of *P*. *aeruginosa* colonization status based on Leeds criteria is also required in the French CF registry. Finally, since the CF airways are colonized by complex polymicrobial communities, it was not possible to better appreciate the role of individual pathogens in disease severity or colonization by *P*. *aeruginosa*.

In conclusion, the current study highlights a number of clinical factors independently associated with the prolonged absence of *P*. *aeruginosa* in CF adults. The lower prevalence of aspergillosis, diabetes, pancreatic insufficiency and F508del/F508del genotype in the “Never” population together with older age at CF diagnosis are consistent with a milder form of the disease and better capacity of this minority of patients to resist colonization by *P*. *aeruginosa* and other environmental microbes. It would be interesting to examine the genetic traits of these patients to identify predictors of *P*. *aeruginosa* colonization according to the CFTR genotype. This would allow a more appropriate monitoring of patients from childhood onwards according to individual risk.
